# Injuries in Mixed Martial Arts After Adoption of the Unified Rules of MMA: A Systematic Review

**DOI:** 10.1177/23259671251342578

**Published:** 2025-07-04

**Authors:** Vilius Zachovajevas, Lars Engebretsen, Gilbert Moatshe, Pavelas Zachovajevas, Olav Røise

**Affiliations:** † Institute of Clinical Medicine, Faculty of Medicine, University of Oslo, Oslo, Norway; ‡ Division of Orthopaedic Surgery, Institute of Clinical Medicine, Oslo University Hospital, Oslo, Norway; § Department of Health Promotion and Rehabilitation, Lithuanian Sports University, Kaunas, Lithuania; ‖ Norwegian Trauma Registry, Division of Orthopedic Surgery, Oslo University Hospital, Oslo, Norway; Investigation performed at University of Oslo, Oslo, Norway

**Keywords:** mixed martial arts, sports medicine, combat sports injuries, athlete safety, injury prevention

## Abstract

**Background:**

The existing literature on injuries in mixed martial arts (MMA) is sparse and lacks a summarized review of injuries in the sport after the adoption of the new rule set in 2009.

**Purpose:**

To assess the injury characteristics in terms of injury rates and types in MMA after the adoption of the Unified Rules of MMA (URM) by the Association of Boxing Commissions and compare them with preadoption trends.

**Study Design:**

Systematic review; Level of evidence, 4.

**Methods:**

Under PRISMA (Preferred Reporting Items for Systematic Reviews and Meta-Analyses) guidelines, primary literature in English examining injuries in MMA after URM adoption was scanned in Medline, Embase, and PubMed from the inception of these databases until December 27, 2023. Reports providing relevant information on injury rates and characteristics, with data solely on MMA athletes rather than in a combination of athletes from other combat sports, were reviewed and analyzed. In total, 191 reports were identified after the initial search. Descriptive statistics were employed to summarize injury rates, types, and locations, as well as variations across subgroups.

**Results:**

A total of 43 reports were included in the analysis. The reports varied widely in design, injury definitions, and data collection methods. Post-URM competition injury rates ranged from 23.6 to 54.5 injuries per 100 athlete-exposures (AEs), with soft tissue injuries (lacerations/abrasions/contusions) being the most common type of injury (20.7%-56.9% of all injuries). The most common location of injury was the head and neck area (29.5%-75.9% of all injuries). Concussion rates varied from 14.7 to 16.1 per 100 AEs, and heavier fighters experienced more knockouts and technical knockouts. There is some evidence suggesting higher injury rates among professional fighters.

**Conclusion:**

The review demonstrated that the most common injuries reported in MMA athletes are soft tissue injuries including lacerations, abrasions, and contusion mainly in the head and neck area. Professional athletes seem to have higher injury rates than amateur athletes, while heavier weight fighters sustain more knockouts and technical knockouts. Current injury rates and types seem to remain similar to those before the adoption of the URM. Analysis of current literature emphasizes a lack of standardized definitions, data on training injuries, and female injuries, which are required to fully evaluate injury characteristics in MMA and ensure the long-term well-being of those participating in the sport.

Mixed martial arts (MMA) is a dynamic combat sport that integrates striking with hands, legs, elbows, and knees; grappling; and ground fighting. Amateur bouts consist of three 3-minute rounds,^30^ while professional competitions consist of 3 or 5 rounds, each lasting 5 minutes, ending by a fighter’s concession, time expiration, or referee/ringside physician stoppage when the fighter is unable to defend properly, such as after losing consciousness.^4^ Gaining global popularity and continuing to grow in number of events and revenue,^23,57^ MMA faced criticism for its perceived violence. As a response, a new ruleset banning multiple dangerous techniques such as attacks on the spine, headbutting, and kicking a grounded opponent was introduced.^28^ This new ruleset, called the Unified Rules of MMA (URM), was adopted by the Association of Boxing Commissions (ABC) in 2009, mandating that all forthcoming professional MMA bouts in North America adhere to the established ruleset, with an aim of enhancing athlete safety.^3^ Although minor changes in ruleset have been made since, allowed fighting techniques remained unchanged until November of 2024 when elbow strikes delivered in a vertical motion became allowed.

As MMA expands, understanding athlete injuries and their sequelae becomes crucial. The diverse study designs, coupled with dynamic sport nature and rule changes, necessitate a comprehensive approach. Even though several reviews on the topic exist^11,41,43,70^, none specifically examines post-URM adoption data, marked by over 30 new rules, drastically altering fight dynamics and potentially injuries sustained by the athletes, leaving a significant knowledge gap on the current injury characteristics and the effectiveness of the new rules.

By synthesizing data on injury prevalence, types, and impacts within the sport, we aimed to explore how current evidence can inform about injury prevention strategies, evidence-based practices, and promote athlete well-being the URM may have had on MMA. The purpose of our study is to address the aforementioned knowledge gap by providing insights into injury patterns and risk factors in contemporary MMA. Through this review, we aim to inform sports medicine professionals, researchers, athletes, coaches, and governing bodies about the nature, frequency, and consequences of injuries in MMA, ultimately contributing to improved evidence-based practices and promotion of athletes’ well-being. Our hypothesis is that the diverse and multifaceted nature of injuries in MMA highlights the need for continued research and refined safety guidelines to better protect athletes while preserving the integrity of the sport.

## Methods

### Literature Search

We conducted a comprehensive search of relevant literature to identify studies related to injuries in MMA. The following databases were systematically searched: Medline, Embase, and PubMed from the inception of these databases until December 27, 2023. The search and report strategy was developed in accordance with the PRISMA (Preferred Reporting Items for Systematic Reviews and Meta-Analyses) guidelines.^55,56,59^

Preliminary searches were initially performed to identify potential keywords and appropriate controlled vocabulary terms. No restrictions were applied based on publication type or status, ensuring a comprehensive review of the available literature. The keywords in the search included variations of words for contusion, hematoma, laceration, abrasion, damage, cut, injury, rupture, fracture, and concussion in reports on martial arts. The detailed search strategy can be found in the Supplemental Material 1 and 2 (available separately). All the search and data collection was carried out by the first author (V.Z.) independently.

### Report Selection

After removing duplicates, records underwent 2-step screening by the first author, who had completed formal literature search and screening courses provided by the library at the affiliated medical faculty. Titles were first screened to exclude irrelevant studies. Remaining records were assessed for eligibility criteria during full-text evaluation.

Because most of the included records were observational studies, we focused on measurements such as rates of general injury incidence, injuries by specific location, injuries by specific type, concussions, size measurements of certain brain structures, and scores for cognitive and neurological testing.

The level of evidence for this systematic review corresponds to level 5 according to the suggestion by Melnyk and Fineout-Overholt.^49^

#### Inclusion Criteria

Non–case report studies were included if they met the following criteria: (1) focus on MMA injuries and risk factors: the report primarily addressed issues, injuries, or risk factors for sustaining injuries within the context of MMA; (2) specific analysis of MMA athletes (when athletes were included in the study), rather than in combination of athletes of other combat sports; (3) relevance to injury types, mechanisms, and prevalence: the report provided pertinent data regarding the types, mechanisms, and prevalence of injuries or issues directly related to MMA; (4) post-2009 data collection: if the period of data gathering was not described, only studies with the publication year of 2011 or later were included; (5) studies available in English: studies with data from amateur, professional, or both amateur and professional fighters were included. Data on both training and competition injuries were reviewed in this study.

#### Exclusion Criterion: Weight Cuts in MMA

Articles assessing injuries and issues specifically related to weight cuts (also referred to as “rapid weight loss” in medical literature) were excluded from the study to maintain a focused analysis on injuries sustained during actual combat and training activities, rather than addressing a distinct aspect of MMA that involves weight manipulation practices. While relevant to the overall MMA landscape, weight cuts present a distinct area of investigation with their own set of complexities and considerations.

#### Exclusion Criterion: Secondary Literature

**Figure 1.**
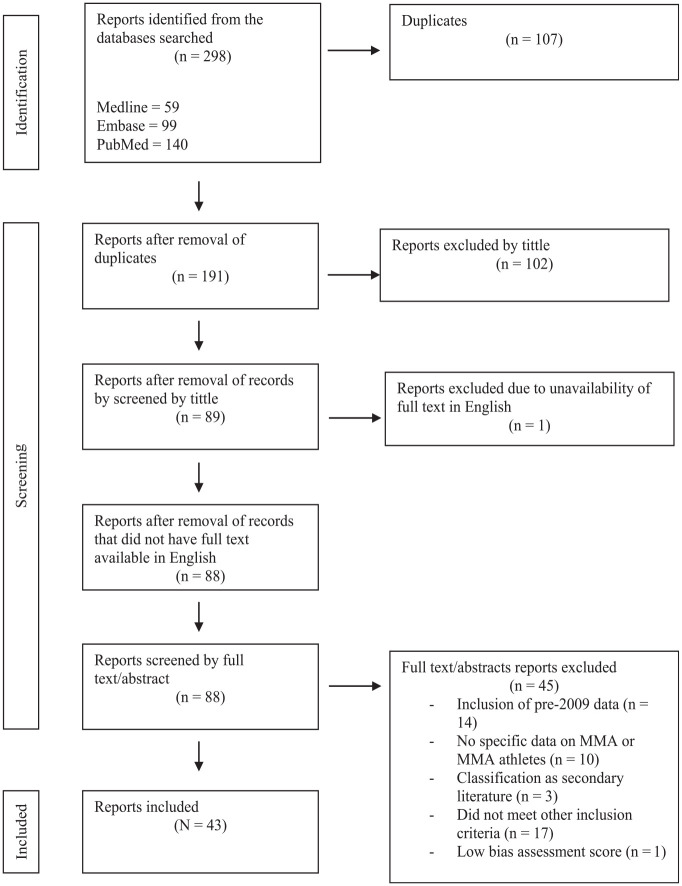
PRISMA (Preferred Reporting Items for Systematic Reviews and Meta-Analyses) flow diagram of identification, screening, and inclusion of reports in the current study. MMA, mixed martial arts.

Secondary literature, such as literature reviews, was excluded from the study to uphold the emphasis on original research that offers direct empirical data. By excluding secondary literature, this study aims to maintain a rigorous examination of primary research, enhancing the reliability and specificity of the findings. Despite direct exclusion, reference lists of secondary literature were screened for additional relevant articles. Summary of report selection is presented in Figure 1.

#### Bias Assessment

The risk of bias for all non–case report studies was evaluated by the first author independently using evaluation criteria based on Saw et al.^62^ Studies were scored on an 8-point scale based on the presence of peer review of the publication, number of participants/simulations, definition of the population, description and replicability of study design, and description of MMA athlete parameters (Appendix Table A1). Studies with a score ≥4 were included. One study with a lower score was excluded because of a high risk of bias (Appendix Table A2).

### Statistical Analysis

Injury rates categorized by location and type were extracted and reported. Where available, the number and percentage of injuries and injury rate per athlete-exposure (AE) are reported. Descriptive statistics were used to categorize injuries by type, location, and severity, as well as to examine variations in injury rates among different subgroups, such as professional versus amateur athletes and weight classes. Due to the heterogeneity of the included studies in terms of methodology, definitions, and reporting, no pooling of data was performed.

## Results

The initial search returned 298 records. After duplicate removal and title/language screening, 88 reports underwent full-text evaluation (Figure 1). A total of 45 reports were excluded for the following reasons: inclusion of pre-2009 data (n = 14), no specific data on MMA or MMA athletes (n = 10), classification as secondary literature (n = 3), did not meet other mentioned inclusion criteria (n = 17). One study was excluded because of low bias assessment score. Ultimately, 43 reports were included in this review.

The majority (27.9%) of studies were of a retrospective cohort design, followed by case reports (23.3%), cross-sectional studies (18.6%), and prospective cohort studies (16.3%). A summary of report designs is provided in Table 1.

**Table 1:** Summary of Report Designs With References

Study Design	Included Report With Reference	Title
Prospective cohort study (n = 8)	Bray et al^10^ (2021)	Effect of weight class on regional brain volume, cognition, and other neuropsychiatric outcomes among professional fighters
	Conway Kleven et al^13^ (2023)	Longitudinal changes in regional brain volumes and cognition of professional fighters with traumatic encephalopathy syndrome
	Coswig et al^14^ (2016)	Time-motion and biological responses in simulated mixed martial arts sparring matches
	Ghoul et al^26^ (2019)	Mixed martial arts induces significant fatigue and muscle damage up to 24 hours postcombat
	Jansen et al^32^ (2021)	Characterizing head impact exposure in men and women during boxing and mixed martial arts
	Kirk and Childs^36^ (2023)	Combat sports as a model for measuring the effects of repeated head impacts on autonomic brain function: a brief report of pilot data
	Mayer et al^47^ (2015)	A longitudinal assessment of structural and chemical alterations in mixed martial arts fighters
	Wiechmann et al^72^ (2016)	Evaluation of muscle damage marker after mixed martial arts matches
Retrospective cohort study (n = 12)	Bernick et al^7^ (2021)	Concussion occurrence and recognition in professional boxing and MMA matches: toward a concussion protocol in combat sports
	Curran-Sills and Abedin^16^ (2018)	Risk factors associated with injury and concussion in sanctioned amateur and professional mixed martial arts bouts in Calgary, Alberta
	Fares et al^19^ (2023)	Upper limb injuries in mixed martial arts
	Fares et al^20^ (2019)	Musculoskeletal and head injuries in the Ultimate Fighting Championship (UFC)
	Fares et al^21^ (2021)	Craniofacial and traumatic brain injuries in mixed martial arts
	Follmer et al^25^ (2019)	Head trauma exposure in mixed martial arts varies according to sex and weight class
	Ji^33^ (2016)	Analysis of injury types for mixed martial arts athletes
	Jones et al^34^ (2023)	Characteristics of facial trauma in professional mixed martial arts
	Khatib et al^35^ (2024)	Brain trauma characteristics for lightweight and heavyweight fighters in professional mixed martial arts
	Ross et al^60^ (2021)	Injury profile of mixed martial arts competitions in the United States
	Ross et al^61^ (2013)	Injury patterns of mixed martial arts athletes in the United States (published only as abstract for presentation)
	Sifuentes-Cervantes et al^66^ (2021)	Maxillofacial trauma in the Ultimate Fighting Championship
Cross-sectional study (n = 8)	Alm^2^ (2014)	The prevalence of concussion in mixed martial arts (published only as abstract for poster presentation)
	Banks et al^5^ (2014)	The protective effect of education on cognition in professional fighters
	Esagoff et al^18^ (2023)	Sparring and the brain: the associations between sparring and regional brain volumes in professional mixed martial arts fighters
	Heath and Callahan^27^ (2013)	Self-reported concussion symptoms and training routines in mixed martial arts athletes
	Neel et al^51^ (2023)	Articulation rate, pauses, and disfluencies in professional fighters: potential speech biomarkers for repetitive head injury
	Scott et al^64^ (2015)	Incidence of pinna haematoma in mixed martial arts (only abstract published)
	Shin et al^65^ (2014)	Diffusion measures indicate fight exposure–related damage to cerebral white matter in boxers and mixed martial arts fighters
	Stephen et al^67^ (2020)	The relationship between fighting style, cognition, and regional brain volume in professional combatants: a preliminary examination using brief neurocognitive measures
Case-control study (n = 3)	Bernick et al^6^ (2015)	Repeated head trauma is associated with smaller thalamic volumes and slower processing speed: the Professional Fighters’ Brain Health Study
	Fogarty et al^24^ (2019)	Head motion predicts transient loss of consciousness in human head trauma: a case-control study of mixed martial artists
	Lockwood et al^41^ (2018)	Traumatic brain injuries in mixed martial arts: a systematic review
Case report study (n = 10)	Bonotto et al^9^ (2016)	Professional karate-do and mixed martial arts fighters present with a high prevalence of temporomandibular disorders
	Chang & Chang^12^ (2021)	Early degenerative joint disease of the elbow in professional fighting: a case series (only abstract published)
	Crilly et al^15^ (2018)	"Mixed" trauma to the carotid artery in a mixed martial arts injury—a case report and review of the literature
	Ferrel et al^22^ (2021)	Constrictive pericarditis in the setting of repeated chest trauma in a mixed martial arts fighter
	Lee et al^39^ (2015)	Omohyoid muscle syndrome in a mixed martial arts athlete: a case report
	Lubner et al^42^ (2019)	Hearing threshold shift after mixed martial arts fights: a field research study (published only as abstract for poster presentation)
	Maerki et al^44^ 2012	Giant cell granuloma of the temporal bone in a mixed martial arts fighter
	Makar et al^45^ (2019)	"Knock out" pancreas: an unusual case of isolated pancreatic duct laceration in a mixed martial arts fighter (only abstract published)
	Meulener and Smith^50^ (2011)	Herpes gladiatorum with ocular involvement in a mixed martial arts fighter
	Stewart et al^68^ (2016)	Ustilago echinata: infection in a mixed martial artist following an open fracture
Simulation/observational study (n = 2)	Tiernan et al^71^ (2021)	Finite element simulation of head impacts in mixed martial arts
	Zhan et al^73^ (2021)	Predictive factors of kinematics in traumatic brain injury from head impacts based on statistical interpretation

### General Overview of Injury Rates

#### Injury Rates in Amateur and Professional MMA Athletes in Competition

Four studies^16,19,20,60^ assessed overall injury rates in MMA, all relying on subjective evaluations by ringside physicians without clear injury definitions (Table 2). Post-2009, overall injury rates ranged from 23.6 to 54.5 injuries per 100 AEs.

**Table 2:** Overall Injury Rates in MMA Competitions After Year 2009*^*a*^*

	Injury Rate per 100 AEs	Method of Assessing Injuries	Definition of Injuries by Physician	Athlete Level
Fares et al^20^	51	Assessment by ringside physician after a bout	No definition provided	Professional
Ross et al^60^	39.9	Assessment by ringside physician after a bout	No definition provided	Amateur and professional
Fares et al^19^	54.5	Assessment by ringside physician after a bout	No definition provided	Professional
Curran-Sills and Abedin^16^	23.6	Assessment by ringside physician after a bout	No definition provided	Amateur and professional

a AE, athlete-exposure; MMA, mixed martial arts.

Both studies comparing athletes by their competition level reported that professionals exhibited higher injury rates than amateurs (59 vs 30.4 injuries/100 AEs in study by Ross et al)^60^; however, the greater odds ratio for sustaining an injury was no longer significant when adjusted to other variables in the study by Curran-Sills and Abedin.^16^ It is, however, important to note that both studies that included amateurs reported notably lower injury rates.

Maxillofacial trauma occurred in 14.5% of Ultimate Fighting Championship (UFC) bouts, while facial injuries comprised 15.8% of all injuries sustained in UFC bouts.^34,66^

#### Injury Rates in Male and Female MMA Athletes

Limited supporting data on sex-based differences suggest that men have higher injury rates (54 vs 30 injuries/100 AEs in women)^20^ and increased likelihood of facial injuries.^20,34^ Maxillofacial injuries were primarily reported in men, although statistical analyses were not described.^66^

#### Other Factors for Injury Rates

The effect of weight class on overall injury rates remains inconclusive, with contradictory findings.^20,61^ Age showed no significant effect on competition injury rates.^61^ Losing a fight was correlated with higher overall injury rates (48% of losers sustained injuries vs 24% of winners), even when amateurs and professionals were compared separately.^60^ Professionals who lost their bouts also sustained more facial injuries.^34^

Fight outcomes, particularly KO/TKO (knockout/technical knockout) and nonsubmission results, were associated with increased overall injuries and longer durations of medical suspension.^16,66^ Stoppage by referee or athlete's corner as well as fight ending in draw or in no contest (ie, a bout that is stopped due to an accidental injury or rule violation, resulting in no official winner) (1 variable) also led to increased rates of overall injuries.^16^ Nonsubmission outcomes were also predisposing factors for facial injuries among professional athletes.^34^

#### Injury Location

**Figure 2.**
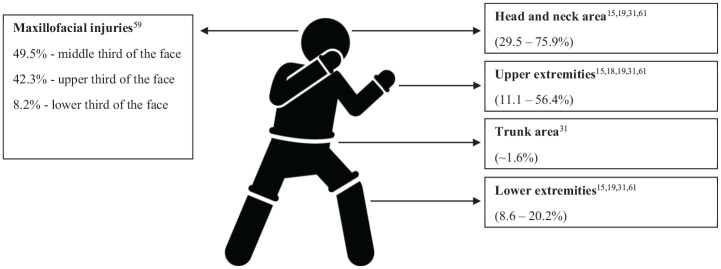
Illustration of injury distribution in mixed martial arts. Image used under purchased license from Depositphotos.

Six studies explored injury distribution by anatomic location^16,19,20,33,61,66^ (Figure 2). Head and neck areas were most susceptible to injuries in competition, ranging from 29.5% to 75.9% of competition injuries and injuries in athletes visiting medical institutions multiple times^16,20,33,61^: 49.5% of maxillofacial injuries occurred in the middle third, 42.3% in the upper third, and 8.2% in the lower third of face among professional MMA fighters in competition.^66^

Injuries to the extremities followed head injuries, with divergence on whether upper (11.1%-56.4%) or lower extremity injuries (8.6%-20.2%) were more dominant^16,19,20,33,61^. Sex-specific differences were suggested, with 1 study reporting significantly higher upper extremity injury rates among women (40% vs 14% of all injuries) and higher lower extremity injury rates among men (19% vs 5% of all injuries),^20^ while another study found no significant difference in upper extremity injury rates between male and female fighters.^19^

Regarding upper extremity injuries, the hand was noted as the most injured anatomic location (66.67% of all upper extremity injuries), primarily caused by striking the opponent (61.7% of all upper extremity injuries) rather than being hit (5%).^19^ Trunk injuries seem to be the least common, accounting for 1.6% of all injuries in MMA athletes visiting medical institutions >3 times.^33^

### Injury Types

#### Injury Types in MMA

Studies on injury types in MMA athletes reported varying findings, making systematization challenging. However, soft tissue injuries (20.7%-56.9%), tendon/ligament injuries (11.2%-23.3%), fractures (6.2%-14%), and altered mental state/concussion (4.5%-20.8%) were consistently among the most common injuries reported in MMA athletes^33,60,61^ (Table 3).

**Table 3:** Overview of the Distribution of Injury Types in MMA*^*a*^*

		Ross et al^60^ (N = 401 Injuries)	Ross et al^61^ (N = 891 Injuries)	Ji^33^ (N = 860 Injuries)	Curran-Sills and Abedin^16^ (N = 162 Injuries)
Injury type	Fractures	12.5	14.0	6.2	5.6
	Tendon/ligament injuries	12.0	**23.3**	11.2	32.1 (grouped together as lacerations + soft tissue injuries and unspecified injuries)
	Concussions/altered mental state	12.2	4.5	20.8	**62.3** (defined as sustaining KO/TKO)
	Soft tissue injuries (lacerations/ abrasions/ contusions)	**56.9**	20.7	**53.8**	32.1 (grouped together as lacerations + soft tissue injuries and unspecified injuries)
	Other	6.4	37.5*^*b*^*	8.0	0.0 (no injuries were categorized as “Other,” but the authors did not explicitly state their absence)
Method for data gathering		Assessment by ringside physician after a bout	Anonymous survey on several MMA sites and forums	Questionnaires for MMA athletes who had visited a medical institution more than thrice and were diagnosed with injuries	Assessment by ringside physician after a bout
Athlete level		Amateur and professional	Amateur and professional	Not specified	Amateur and professional

a Data are reported as percentages. Bolded percentages indicate the most common injury group in each study. KO/TKO, knockout/technical knockout; MMA, mixed martial arts.

b Even though it might appear that the “Other” group is the largest, it is a mash of multiple different injury types, as authors specifically state that the group of sprains/strains is the largest, with 23.3% of all injuries.

In 1 study, concussions were reported as the most common overall injury type,^16^ although the study defined concussion as sustaining KO/TKO, potentially inflating concussion rates significantly and affecting the overall injury distribution.^52^

#### Injury Types Among Amateur and Professional MMA Athletes in Competition

One study looked into injury types among professional and amateur MMA competitors separately and reported slight differences: professionals sustained significantly more lacerations and bone fractures or cartilage damage (38.6% and 15.7%, respectively, of all injuries among professionals) than amateurs (22.6% and 9.3%, respectively, of all injuries among amateurs).^60^ Amateur MMA fighters, however, were significantly more likely to sustain epistaxis, concussions, and contusions or hematomas when compared with professional MMA fighters (7.8%, 15.7%, and 30.9% vs 1.5%, 8.6%, and 21.8%, respectively, in respective levels of competition).^60^

#### Injury Types of Specific Injuries in MMA

Lacerations were also the most common facial injury (12%), while fractures were less common (3.6%) and located primarily in the nasal or orbital bones.^34,66^ Maxillofacial injuries in professionals showed lacerations dominating the injuries of the upper third of the face (100%), while middle-third injuries consisted of lacerations (40.5%) and fractures (59.5%) in orbital (40.7%), nasal (38.8%), and zygomatic bones (7.4%).^66^ Lower-third injuries included mandibular fractures (53.85%) and lacerations (46.15%).^66^

**Figure 3.**
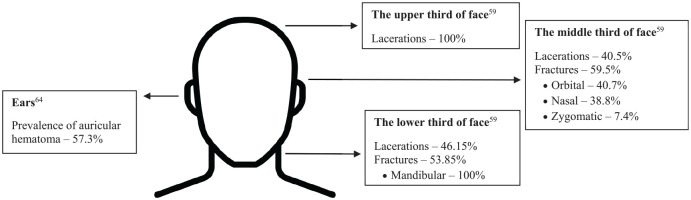
Overview of facial injuries in MMA athletes. Image used from EduZabawy.com.

"Cauliflower ear" (auricular hematoma) was prevalent, with 57.3% of participants from an online forum reporting its occurrence and 24.9% stopping training because of it.^64^ Concerns about appearance (17.3%) and perceived permanence (58.8%) were also reported.^64^ Summary of findings on facial injuries in MMA is presented in Figure 3.

Three studies explored muscle damage biomarkers in MMA athletes.^14,26,72^ Creatine kinase levels increased 24 to 48 hours after a fight or sparring in all but 1 study.^26^ Myoglobin (Mb) rose significantly in 1 study^72^ 2 hours after a bout, with the majority of Mb variation (87%) correlating with fight duration and the number of obtained hits to the upper and lower body.^72^ This suggests that microscopic muscle damage in MMA involves exercise-induced metabolic stress as well as obtained hits to the muscle tissue.

Lactate increased immediately after sparring, returning to normal values in 48 hours.^14^ Lactate dehydrogenase rose significantly during and after the bout, but sustained muscle damage had a limited effect on physical performance, as only the countermovement jump values were significantly lower in athletes 30 minutes after the bout.^26^ These values have been shown to return to resting levels 24 hours after the bout, and no significant changes in other performance tests were observed.^26^ Similarly, another study, despite increased creatine kinase, found no differences in various performance measures before, immediately after, and 48 hours after sparring.^14^

### Head Impacts in MMA

Fares et al^21^ concluded that traumatic brain injuries (TBIs) may constitute up to 46% of head injuries in MMA. In MMA training, 1 study recorded a mean of 15.7 significant head impacts per sparring session, with no reported injuries during training.^71^

Characterizing head impact exposure, there seems to be no significant sex difference in impact magnitude,^32^ but lightweight fighters sustained more head punches than heavyweights.^35^ Impact distribution in MMA was more evenly spread than in boxing.^32^ Conway Kleven et al^13^ found no significantly increased prevalence of traumatic encephalopathy syndrome in professional MMA fighters.

### Concussions in MMA

#### General Concussion Rates

Six studies^2,7,16,21,25,27^ evaluated concussion rates in MMA, but different definitions for concussion were used. One retrospective study demonstrated that 34% athletes self-reported KO or TKO experiences,^27^ while about 26% of MMA athletes reported receiving a concussion diagnosis from a health care professional over the course of their career.^2^

TBI rates appear to be similar in exclusively professional (16.1/100 AEs) and combined professional and amateur populations (14.7/100 AEs) in competition, with neither having significant sex differences.^16,21^ Professionals had higher KO/TKO rates in the heaviest male divisions.^25^ Video analysis of professional competition suggests about 1 concussion per 11.76 minutes of fight time.^7^

#### Mechanism of Sustaining Concussion

Studies suggest that head angular velocity,^73^ acceleration,^71^ and motion^24^ play roles in sustaining TBI. Most head injuries occur during stand-up striking, with punches being the most common cause.^21^

### Structural Brain Alterations in MMA Athletes

Seven studies have assessed structural brain alterations with magnetic resonance imaging in MMA athletes^6,10,18,40,47,65,67^ and an association between structural brain changes and general participation in MMA,^40,47^ number of sustained Kos,^65^ weight class,^10^ fighting style,^6,67^ number of fights^6,65^, intensity of exposure,^6^ years of training,^6^ and number of sparring practice rounds per week^18^ has been reported.

### Cognitive and Neurological Impairments in MMA Athletes

Studies on cognition showed mixed results, with MMA athletes presenting lower scores in memory,^47^ processing speed,^47^ and articulation rates^51^ than controls, but performing better than boxers in certain cognitive domains.^67^

An increase in the neuroptics proprietary variable after MMA sparring suggests that repeated head impacts, even during training, may disrupt autonomic brain function without outwardly visible symptoms.^36^

A subtle decrease in reaction time scores for MMA participants with ≤12 years of education compared with those with higher levels^5^ suggests an association with minimal effect on overall performance.

### Other Reported Injuries and Issues

Ten case reports on the topic exist, spanning from acute injuries sustained directly in MMA (such as omohyoid muscle syndrome,^39^ fracture of the superior horn of the thyroid cartilage with a carotid artery pseudoaneurysm,^15^ acute pancreatitis,^45^ fungal skin infections,^68^ hearing threshold shift^42^) to more chronic illness postulated to be related to MMA (such as giant cell granuloma within the temporal bone,^44^ herpes simplex virus type 1,^50^ constrictive pericarditis,^22^ ostheoarthritis,^12^ and temporomandibular disorders^9^).

## Discussion

The major findings of our review highlight the complex health challenges faced by MMA athletes, revealing the variety of injuries that occur both in competition and in training. The high incidence of traumatic injuries, such as soft tissue damage and fractures, combined with such chronic issues as osteoarthritis and neurological impacts, suggests that both short-term and long-term health risks are significant. Notably, the findings on repetitive head trauma and its association with chronic neurological conditions should prompt further investigation into protective measures and guidelines for head impacts. Additionally, the variation in injury prevalence between different subgroups (eg, professional vs amateur athletes) suggests that more targeted prevention strategies may be necessary. Understanding the multifaceted nature of these injuries is crucial for developing training modifications and rule adaptations aimed at reducing harm while preserving the competitiveness of the sport. It is also important to note that this review analyzed reports with data after the adoption of the URM, and even though not all injuries depend on the changing of the rules (such as pericarditis or skin infections) and are expected to mostly affect competition injuries, all of the reported injuries not meeting the exclusion criteria were included, as the aim of this review was to provide a full picture of the injury landscape in MMA, both in and out of competition.

The interval of competition injury rates provided in this study seems to be comparable with competition injury rates in boxing,^46^ is higher than that in Brazilian jiu-jitsu,^37^ and wrestling,^1^ and slightly lower than that in Muay Thai.^69^ This illustrates that combat sports relying on striking lead to higher incidence of injury, and combat sports relying on grappling lead to lower incidence of injury, while MMA—a combination of both striking and grappling—might find itself somewhere between the 2.

Our review found major variations in reported numbers and drawn conclusions within the literature of MMA injuries. For example, the reported spectrum of overall competition injury rates of 23.6 to 54.5 injuries/100 AEs includes or is close to previously reported weighted means in reviews including pre-URM data (22.9 injuries/100 AEs [men and women] and 24.6 injuries/100 AEs [men]^43,70^ and reported injury rates in studies with data before the adoption of the URM (23.6, 23.7, and 28.6 injuries/100 AEs)^8,53,63^; however, the span of it raises a question as to the underlying reason for such variation. As discussed earlier in the review, even though injury rates may vary from objective factors such as athlete level, percentage of female participants, or weight classes included, subjective factors, such as individual examination by a single ringside physician and subjective definitions of injuries, might have also contributed to the scattering of reported numbers.

Another prominent factor contributing to the divergent findings is the lack of standardized data collection methods across studies. This could be illustrated by the variation in, for example, reported distribution of injury types (Table 3), where 2 of 4 mentioned studies used surveys for athletes, while the rest of the studies gathered data from physician reports. Athletes’ recall bias and subjective opinion on what was considered an injury might have played a significant role in the information provided and therefore the variation of injuries. The absence of a consensus on the definition of injury in studies on MMA is another layer to this methodological challenge, not only on the participant but also on the researcher level. The interpretation of what constitutes a significant injury varies among researchers, physicians, and athletes. A minor cut or abrasion might be classified as a notable injury in one study, while in another an athlete might only report more impactful events such as fractures or concussions. This inconsistency in defining injury introduces a substantial source of heterogeneity, making it arduous to compare and aggregate findings across studies.

The dominance of soft tissue injuries^48,53,54^ and injuries to the head and neck area^53,58^ was already reported in studies that analyzed data before 2009 and thus before adoption of the URM, which suggests minimal changes in injury types. However, a study by McClain et al^48^ that used data from 2008 to 2012 reported a notably lower injury rate than previously discussed: 8.5 injuries per 100 AEs. This finding sounds counterintuitive, as it is unlikely that inclusion of more rules protecting the athletes would lead to higher injury rates, and it could potentially be attributed to the inclusion of both amateurs and professionals, as well as absence of the definition of "injury" in the study or the possibility of “overprotection” of athletes by the referees in the transition period right after the new rules were adopted. This could be further supported by the injury rate of 23.6 injuries per 100 AEs among professional MMA athletes reported in a study with data from 2002 to 2007,^53^ 23.7 among professional men from 1999 to 2006,^63^ and 28.6 among professional men from 2001 to 2004.^8^ These findings suggest that the injury rates in modern MMA are similar to or possibly higher than those in pre-URM MMA; however, it is difficult to draw certain conclusions because of the earlier discussed limitations in the existing research.

### Implications for Practice, Policy, and Future Research

The identified injuries have significant implications for practice, policy, and future research. Practitioners and policy makers should consider the diverse array of health issues when formulating safety guidelines and regulations for MMA competitions. Enhanced medical screening, thorough prefight evaluations, and comprehensive monitoring during and after bouts are crucial components of athlete safety. Addressing the potential underreporting or misclassification of injuries due to variation in reporting standards is another facet that merits policy attention. Standardizing injury and concussion definitions (eg, as suggested by the International Olympic Committee Injury and Illness Epidemiology Consensus Group^31^) and reporting mechanisms (such as by using previously suggested tools such as MMA−Knockout Tool^38^ and the Sport Concussion Assessment Tool–6^17^) across MMA organizations and studies ensures consistency and facilitates more accurate surveillance, which will in turn enhance the comprehensiveness of injury databases and contribute to a more robust evidence base.

The findings also underscore the need for continued research in several areas. There is a lack of prospective studies, as only 8 of the included reports were of longitudinal design.^10,13,14,26,32,36,47,72^ Even though parameters such as competition injury rates are straightforward to map out, longitudinal studies with larger and more diverse cohorts are essential to better understand the etiology of injuries, their risk factors, and long-term effects on athletes.

Among the reports using athlete data, only 10 included populations with ≥10% female athletes,^10,13,18-21,32,47,67,71^ 10 did not specify female inclusion,^¶^ and 5 did not include any women.^24,42,51,65,72^ This may have skewed the results, as multiple studies found significantly different injury rates between male and female athletes,^20,34^ highlighting the need for greater female inclusion in future research. Other suggestions that should be implemented in future research include exploring the differences within the professional and amateur groups separately, such as mapping injuries and injury rates in relation to weight, age, or athletes’ experience, as well as exploring training injuries separately.

One critical gap in the current literature is the lack of focus on time lost from sport due to injury. Simply reporting the number or types of injury without considering how long an athlete is out of training or competition does not provide a complete understanding of injury severity or impact. Time out of sport is a crucial metric that reflects the true burden of an injury on an athlete’s career, training progress, and overall well-being. Future research should not only document the type and location of injuries but also analyze how these factors correlate with recovery time. This would offer a more comprehensive picture of injury consequences and could inform more effective prevention and rehabilitation strategies.

Another issue is lack of studies on training injuries. One study published in the same year that the URM was adopted by ABC reported that 77.9% of self-reported MMA injuries were sustained during training,^58^ similar to a rate of 85.5% of injuries sustained in Brazilian jiu-jitsu.^29^ Therefore, looking just at the injuries sustained in competition might reveal a small part of the injury characteristics in the sport. Additionally, combining training and competition injuries without analyzing them separately obscures important distinctions in injury patterns, mechanisms, and frequencies. This can lead to inaccurate conclusions, as the risk factors, intensity, and nature of injuries sustained during competition may differ significantly from those experienced in training. Thus, studies that clearly differentiate between training and competition injuries are essential for a full understanding of injury etiology in MMA.

### Limitations

The review itself is subject to inherent limitations. The possibility of publication bias, wherein studies reporting more severe or unusual cases are more likely to be published, may skew the overall representation of injuries and psychoneurological effects in MMA. The inclusion of only English-language studies might introduce language bias, excluding potentially relevant non-English literature. A significant limitation in review of the current literature is the lack of standardized injury definition or provision of injury definition used in study, as it contributes to variability in reported injury rates and complicates comparisons across studies. Another notable constraint in the review processes is the fact that the search for reports was carried out by a single author. While efforts were made to meticulously design and execute the search strategy, the absence of a multi-author assessment of bias introduces the possibility of oversight or bias in the selection of studies.

## Conclusion

Our review demonstrated that the most common injuries reported in MMA athletes are soft tissue injuries including lacerations, abrasions, and contusion mainly in the head and neck area. Professional athletes seem to have higher injury rates than amateur athletes, while heavier athletes sustain more KOs and TKOs. Current injury rates and types seem to remain similar to those before the adoption of the URM. Analysis of current literature emphasizes a lack of standardized definitions, data on training injuries and female injuries, which are required to fully evaluate injury characteristics in MMA and ensure the long-term well-being of those participating in the sport.

## Supplemental Material

sj-docx-1-ojs-10.1177_23259671251342578 – Supplemental material for Injuries in Mixed Martial Arts After Adoption of the Unified Rules of MMA: A Systematic Review Supplemental material, sj-docx-1-ojs-10.1177_23259671251342578 for Injuries in Mixed Martial Arts After Adoption of the Unified Rules of MMA: A Systematic Review by Vilius Zachovajevas, Lars Engebretsen, Gilbert Moatshe, Pavelas Zachovajevas and Olav Røise in Orthopaedic Journal of Sports Medicine

## Footnotes

¶ References 6, 7, 9, 14, 27, 35, 36, 60, 64, 66.

## Appendix

**Table A1:** Risk-of-Bias Assessment Score Criteria*^*a*^*

Criteria		Definition	Scoring		
#	Description		0	1	2
1	Peer reviewed	Study published in peer-reviewed journal	No	Yes	−
2	Number of participants	Number of participants/simulations included in study findings	<5	6-30	>31
3	Population defined	Age, sex, sport, experience (or level) was described	No	Partly	Yes
4	Experimental design	Experimental design of the study period was described and replicable	No	Partly	Yes
5	MMA athlete information	MMA athlete parameters were described	No	Yes	−

a MMA, mixed martial arts. Dashes indicate that a score of 2 was not applicable for the corresponding criterion.

**Table A2:** Risk-of-Bias Assessment of the Non–Case Report Studies

Criteria No.	1	2	3	4	5	Total
Study						
Fares et al, 2019	1	2	2	2	1	8
Ross et al, 2021	1	2	1	2	1	7
Fares et al, 2023	1	2	1	2	1	7
Curran-Sills and Abedin, 2018	1	2	2	2	1	8
Sifuentes-Cervantes et al, 2021	1	2	1	2	1	7
Jones et al, 2023	1	2	2	2	1	8
Ross et al, 2013	1	2	1	1	1	6
Ji, 2016	1	2	2	1	1	7
Scott et al, 2015	1	2	0	1	0	4
Wiechmann et al, 2016	1	1	2	2	1	7
Ghoul et al, 2019	1	1	2	2	1	7
Coswig et al, 2016	1	1	1	2	1	6
Fares et al, 2021	1	2	1	2	1	7
Tiernan et al, 2021	1	1	1	1	1	5
Jansen et al, 2021	1	1	1	1	1	5
Khatib et al, 2024	1	2	1	1	1	6
Conway Kleven et al, 2023	1	2	1	1	0	5
Heath and Callahan, 2013	1	2	2	1	1	7
Alm, 2014	1	2	1	1	1	6
Follmer et al, 2019	1	2	1	1	1	6
Bernick et al, 2021	1	1	1	1	1	5
Zhan et al, 2021	1	2	0	2	0	5
Fogarty et al, 2019	1	2	2	2	1	8
Mayer et al, 2015	1	1	1	2	1	6
Lee et al, 2017	1	2	1	2	0	6
Shin et al, 2014	1	2	1	1	1	6
Bray et al, 2021	1	2	2	2	1	8
Bernick et al, 2015	1	2	1	2	1	7
Stephen et al, 2020	1	2	1	2	1	7
Esagoff et al, 2023	1	2	2	1	1	7
Neel et al, 2023	1	2	2	1	1	7
Kirk et al, 2023	1	1	1	2	1	6
Banks et al, 2014	1	2	2	1	1	7
Bickley et al, 2023	1	0 (not specified for MMA)	0	1	0	2
