# Supplementary material for: Injuries in Mixed Martial Arts After Adoption of the Unified Rules of MMA: A Systematic Review
Source: Orthop J Sports Med. 2025 Jul 4;13(7):23259671251342578. doi: 10.1177/23259671251342578 (PMC12227934; doi:10.1177/23259671251342578)
Supplement: sj-docx-1-ojs-10.1177_23259671251342578 – Supplemental material for Injuries in Mixed Martial Arts After Adoption of the Unified Rules of MMA: A Systematic Review [file sj-docx-1-ojs-10.1177_23259671251342578.docx]

# Appendices

### Appendix 1 – Search strategy for injuries in MMA in MEDLINE and EMBASE databases

Search in MEDLINE database includes results from 1946 to December 27 2023; Search in EMBASE database includes results from 1947 to December 27 2023:

| **#** | **Searches** | **Results**  **Medline/Embase** |
| --- | --- | --- |
| 1 | combat sport/ or boxing/ or kickboxing/ or martial art/ or wrestling/ | 3823 / 5633 |
| 2 | (MMA and martial art*).mp. | 167 / 172 |
| 3 | Athletic Injuries/ | 31209 / 26594 |
| 4 | (contusion* or hematoma* or laceration* or abrasion* or damage or cut* or injur* or rupture* or fracture* or concuss*).mp. | 2918641 / 4271771 |
| 5 | 3 or 4 | 2918641 / 4271771 |
| 6 | 1 and 2 | 109 / 160 |
| 7 | 5 and 6 | 59 / 99 |

Search history link for search in Ovid MEDLINE(R) ALL database: http://ovidsp.ovid.com/ovidweb.cgi?T=JS&NEWS=N&PAGE=main&SHAREDSEARCHID=DexBICbxGemnnZUMNP2OjiZlJ010KDP6WXWf8u3D430Z5MIjpH6Iwc0fNwNIG6Q6

Search history link for search in Embase Classic+Embase database: https://ovidsp.ovid.com/ovidweb.cgi?T=JS&NEWS=N&PAGE=main&SHAREDSEARCHID=3OYgcFOqrMdW0JKj5QD1IOu22d5pByONzIrPboD6GeKOwOM9dhFX5XLu9tIHCRpBp

### Appendix 2 – Search strategy for injuries in MMA in Pubmed database

Search in Pubmed database includes results from 2005 to December 27 2023:

| **#** | **Searches** | **Results** |
| --- | --- | --- |
| 1 | (mixed martial arts) AND (injuries) | 140 |

Search history link for search in Pubmed database: https://pubmed.ncbi.nlm.nih.gov/?term=(mixed%20martial%20arts)%20AND%20(injuries)
